# Pulmonary adenocarcinoma associated with Guillain–Barré syndrome: A case report

**DOI:** 10.1097/MD.0000000000010737

**Published:** 2018-05-25

**Authors:** Yuhuan Wang, Sijia Yang, Liang Fang, Yang Liu, Gang Jiang, Xiaoyan Ding, He Wei, Min Liu

**Affiliations:** a Department of Neurology, The Affiliated Hospital of Medical College, Qingdao University, Qingdao, Shandong; b Department of Thoracic Surgery, The First Affiliated Hospital of Zhejiang University, Hangzhou, Zhejiang; c Department of Gastroenterology, The Affiliated Hospital of Medical College, Qingdao University, Qingdao, Shandong, China; d Division of General Internal Medicine, Icahn School of Medicine at Mount Sinai, New York, NY; e Department of Radiology, The Affiliated Hospital of Medical College, Qingdao University; f Department of Pathology, The Affiliated Hospital of Medical College, Qingdao University, Qingdao, Shandong, China.

**Keywords:** Guillain–Barré syndrome, paraneoplastic neurological syndromes, pulmonary adenocarcinoma

## Abstract

**Rationale:**

Guillain-Barré Syndrome (GBS) as a paraneoplastic manifestation of small cell lung cancer has been published several times, while paraneoplastic GBS accompanied by pulmonary adenocarcinoma is rare.

**Patient concerns:**

An 80-year-old male was hospitalized with a 2-week history of fever and 10-day history of progressive ascending muscle weakness in the legs and arms. The patient felt weakness in legs at first when he was still able to move around, but the symptoms gradually progressed to the arms. At the time of office visit, he could no longer walk or hold up objects, and had absent deep-tendon reflexes as well as weakened left lung breath sounds.

**Diagnoses:**

Confirmed by the Cerebrospinal fluid (CSF) and electromyography examination, the patient was originally admitted into our hospital for GBS. However, radiology and histological examination revealed pulmonary adenocarcinoma. He was relatively old and confirmed to have pulmonary adenocarcinoma with simultaneously detected GBS, so was considered to be a paraneoplastic syndrome, rather than pure GBS.

**Interventions:**

The patient was treated with methylprednisolone at 80 mg Qd for 10 consecutive days, which resulted in improvement in arms, then tapering to lower doses for 3 months.

**Outcomes:**

The patient showed temporary relief until relapse 6 months later, when the patient gave up treatment.

**Lessons:**

To our knowledge, this is the first case of pulmonary adenocarcinoma that was diagnosed based on Guillain-Barré-like syndrome, which is very difficult to diagnose and treat. We suggest that elderly patients with GBS should not be considered as simple GBS and should be thoroughly examined to exclude systemic diseases, especially paraneoplastic neurological syndromes. In addition, the elderly should be screened regularly for tumor markers.

## 1 Introduction

Paraneoplastic neurological syndromes (PNS) are neurological diseases related to long-term effects of cancer, not caused by the tumor itself or its metastasis, infection, ischemia, or metabolic disorders. They can affect the central nervous system, peripheral nervous system, neuromuscular junction, or muscle, leading to various neurological symptoms.^[1]^ PNS are relatively common among all cancer patients, with an incident rate <1%,^[2]^ and are more frequently associated with lung cancer.^[3]^ Among lung cancer patients, up to 3% to 5% of patients with small cell lung cancer (SCLC) are associated with PNS.^[4]^ But PNS rarely occur in adenocarcinoma, and even less frequently involves the peripheral nerve. To our knowledge, Guillain–Barré syndrome (GBS) as a paraneoplastic manifestation of SCLC has been published 7 times previously,^[5]^ yet there is no report on pulmonary adenocarcinoma GBS. Therefore, we present the first case of pulmonary adenocarcinoma that was diagnosed based on Guillain–Barré-like syndrome.

## 2 Case presentation

An 80-year-old male presented with a 2-week history of fever and 10-day history of a progressive ascending muscle weakness in the legs and arms. He had smoked 20 cigarettes per day for 55 years. There was a medical history of hypertension. He had a fever and started to cough 2 weeks prior to visit, and took common cold medications on his own. In the past 10 days, the patient felt weakness in legs in the beginning when he was still able to move around. However, his status worsened. He experienced increasing difficulty walking upstairs, standing up, and sitting down. Three days ago, symptoms gradually showed up in the arms, which could not move with ease. At the time of office visit, he could no longer walk or hold up objects.

The physical examination revealed paralysis of the arms and legs (Medical Research Council [MRC] grade 2) with absent deep-tendon reflexes. Sensory examination including light touch, pinprick, vibration, and joint position were all normal. The left lung breath sounds were weakened. His blood pressure was 180/100 mm Hg. The patient reported no difficulty with defecation or urination, but significant weight loss of approximately 3 kg over the last 2 months.

Routine laboratory data showed that urinalysis and fecal tests were normal; levels of autoantibodies such as extractable nuclear antibody spectrum, antiphospholipid antibodies, and antineutrophil cytoplasmic antibodies were normal; blood routine showed a leukocyte level of 20.55 × 10^9^/L, and neutrophil percentage of 88.70; C-reactive protein (70.74 mg/L) and erythrocyte sedimentation rate (60 mm/h) were increased; antinuclear antibodies (ANAs, titer: 1:10,000) were positive; tumor marker examination indicated that carcinoembryonic antigen (CEA, 16.75 ng/mL), neuron-specific enolase (NSE, 28.45 ng/mL), and cytokeratin 19 fragment (CYFRA21-1, 73.96 ng/mL) were elevated; a lumbar puncture showed elevated protein content (1207.00 mg/L), with normal cell count, glucose and chloride levels, and without abnormalities in microbiological cultures or cytology; and the antiganglioside M1 (GM1) IgG antibodies in cerebrospinal fluid (CSF) was positive.

Electromyography examination revealed a significant slowdown of motor nerve conduction velocity in upper and lower extremities, notably decreased amplitude. Nerve conduction studies showed normal sensory studies in the upper and lower extremities, except for the superficial peroneal nerve where the value was undetectable. The left median nerve had prolonged F wave, while the left ulnar nerve, peroneal nerve, and tibial nerve F wave cannot be measured. Spontaneous potential was seen in the legs and arms with reduced recruitment, which suggested severe peripheral nerve demyelination (Table 1). The results for repeated electrical stimulation including low frequency attenuation and high frequency incremental tests were both negative.

**Table 1:**
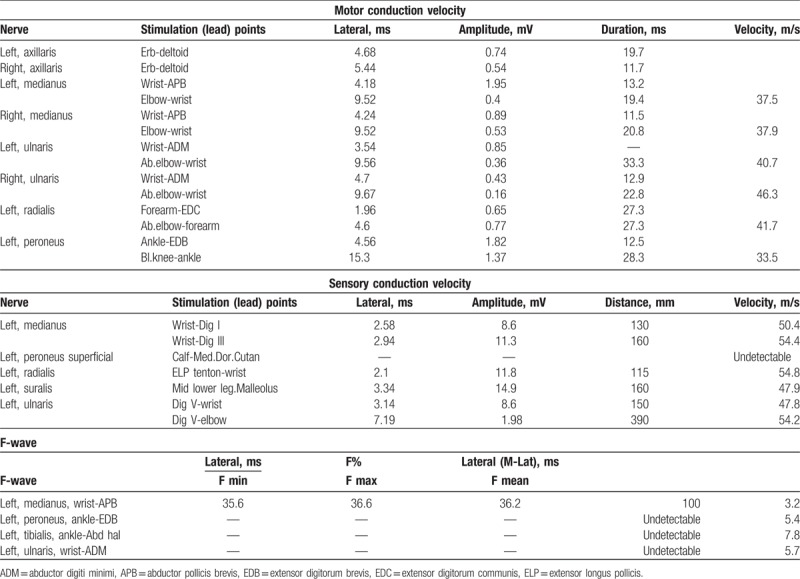
Electromyography examination results.

**Figure 1:**
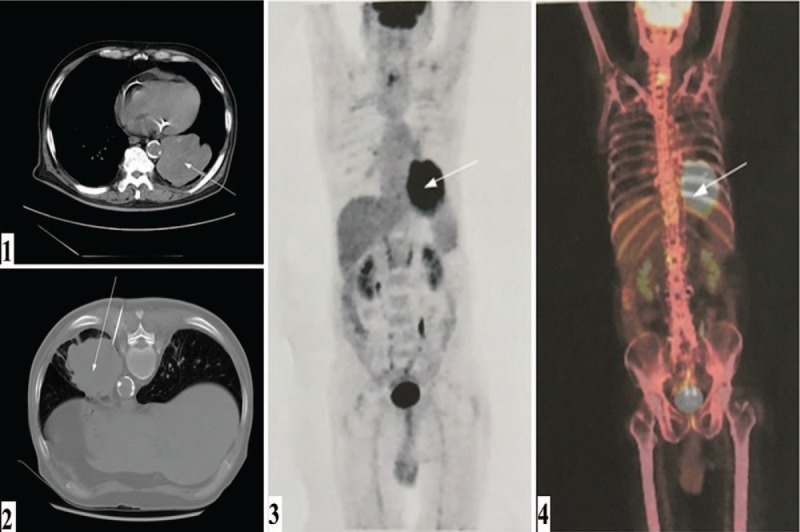
The patient's imaging and pathological findings. Thoracic enhanced computed tomography scan (1, 2). Positron emission tomography-computed tomography imaging (3, 4).

**Figure 2:**
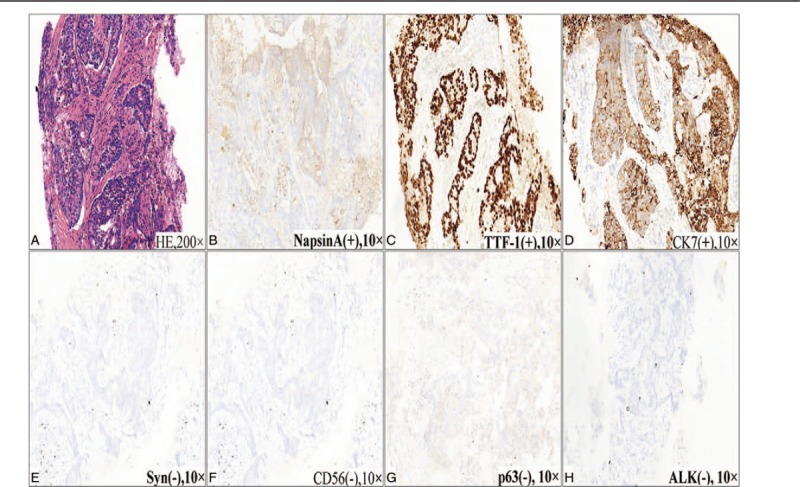
The patient's pathological findings. HE staining showed an infiltrating tubuloglandular proliferation of the tumor, and the tumor cell nucleus was prominent with hyperchromatism, pleomorphism, and pathological mitotic figures (A). Immunohistochemical staining indicated that the tumor cells were positive for NapsinA, TTF-1, and CK, but negative for Syn, CD56, and p63, along with a negative expression of ALK (B–H). ALK = anaplastic lymphoma kinase, CD = cluster of differentiation, CK = cytokeratin, HE = hematoxylin–eosin, Syn = synaptophysin, TTF-1 = thyroid transcription factor-1.

Cerebral magnetic resonance imaging was unremarkable. Thoracic enhanced computed tomography (CT) scan revealed a large mass in the inferior lobe of the left lung; lobulation and spiculation sign were observed in the margin (Fig. 1) that consistent with lung cancer performance. Furthermore, the whole-body positron emission tomography-CT imaging indicated a 69 × 88 mm soft tissue density lumps with fluorodeoxyglucose uptake (maximum standardized uptake value 10.1) in the left inferior lobe without any other abnormal fluorodeoxyglucose uptake (Fig. 1). The CT-guided percutaneous pulmonary biopsy confirmed the diagnosis of poorly differentiated adenocarcinoma. Microscopic examination revealed an infiltrating tubuloglandular proliferation of the tumor, and the tumor cell nucleus was prominent with hyperchromatism, pleomorphism, and pathological mitotic figures. Immunohistochemical staining indicated that the tumor cells were positive for NapsinA, thyroid transcription factor-1, and cytokeratin, but negative for synaptophysin, cluster of differentiation (CD)56, and p63, along with a negative expression of anaplastic lymphoma kinase (Fig. 2).

Based on the test results and clinical manifestation, the final diagnosis was “pulmonary adenocarcinoma and paraneoplastic GBS.” He was treated with methylprednisolone at 80 mg Qd for 10 consecutive days, which only resulted in improvement in arms (MRC grade 4), and the symptoms of the legs did not improve (MRC grade 2). The dose was then adjusted to 40 mg Qd for another 10 consecutive days, followed by oral prednisone 10 mg Qd for 3 months. Given his age and poor general condition, although there was no metastasis, the patient and his family members still chose to discharged from hospital automatically without any surgery or chemotherapy. A follow up was performed 3 months after his discharge, and his symptoms did not worsen. However, the patient experienced systemic weakness again after 6 months; he became completely bedridden and gave up re-admission treatment (Table 2).

**Table 2:**
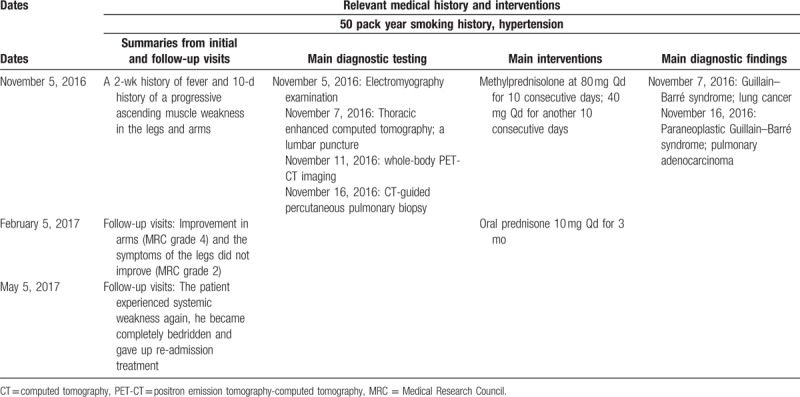
Timeline of interventions and outcomes.

## 3 Discussion

GBS is an acute immune-mediated peripheral nerve dysfunction that is usually provoked by an antecedent infection, such as *Campylobacter jejunitis* or influenza, which was characterized by ascending motor weakness of the extremities that can ascend to the diaphragm, up to 20% of patients develop severe disability and approximately 5% die.^[6]^ In this case, the patient exhibited progressive bilateral and relatively symmetric weakness of the limbs, elevated protein and anti-GM1 IgG antibodies in CSF, and peripheral nerve severe demyelination, which fulfilled the criteria for GBS.^[7]^ Paraneoplastic peripheral neuropathy main lesions is subacute sensory neuropathy. And sensory motor neuropathy, such as GBS, is less frequently observed.^[8]^ Graus et al^[9]^ suggested a diagnostic criteria for PNS: the presence of cancer, the classical syndrome, and well characterized onconeural antibody. In this case, despite the absence of onconeural antibodies, our patient was relatively old and confirmed to have pulmonary adenocarcinoma with simultaneously detected GBS, and so was considered to be a paraneoplastic syndrome, rather than pure GBS.

The exact mechanism of PNS is not well known. PNS is considered to involve an immune response, including severe cross-immunoreaction directed at the tumor and peripheral nerves.^[10]^ Our patient had definite pulmonary adenocarcinoma, so the testing for onconeural antibodies was not carried out. After all, not all patients have well characterized antibodies of PNS, and patients with atypical syndromes or undetected cancer are common.^[2]^ However, he exhibited a positive titer for anti-GM1 IgG antibodies. It may be associated with the immune disorder caused by lung cancer. One research showed that gangliosides may represent onconeural antigens in patients with PNS, whose expression in neoplastic tissue may elicit autoimmune responses against neural structures.^[11]^ The reported case may support this hypothesis.

Researchers have confirmed the presence of ANAs in patients with malignant diseases several decades ago.^[12]^ Large sample, controlled studies of cancer patients’ serum and noncancer subjects’, showed that ANAs were highly elevated in cancer patients.^[13]^ Our patient presented a high ANA titers of 1:10,000; we believed that the ANA may be related to lung cancer. Although the clinical significance of cancer-associated positive ANAs is known little, it seems that ANAs occur more frequently in cancer patients with musculoskeletal symptoms and connective tissue paraneoplastic syndromes.^[13]^ According to this case, for patients with positive ANA results, unless there is evidence for connective tissue disease, the association with PNS should be considered.

CEA and CYFRA are considered as reliable markers of chemotherapy for non-SCLC.^[14]^ Both CEA and CYFRA levels increased in our patient. However, NSE—the most valuable serum tumor marker for SCLC patients—also increased in this case. Therefore, further studies are required to determine the significance of NSE.

There are 2 current, nonspecific approaches to manage PNS, treating the potential tumor to remove antigen source and inhibiting the immune response.^[15]^ Timely and effective treatment of primary tumors is the best way to stabilize PNS. And immunoregulation by corticosteroid, immunosuppressant, anti-CD20 monoclonal antibodies, intravenous immunoglobulin, and plasma exchange constitutes an important part of the treatment.^[1]^ In this case, the patient gave up surgery and chemotherapy. Notably, treatment with methylprednisolone was effective, which further supported the immunological cause.

In conclusion, we herein report the first case of an 80-year-old man who developed pulmonary adenocarcinoma GBS, which is very difficult to diagnose and treat. We suggest that elderly patients with GBS should not be considered as simple GBS and should be thoroughly examined to exclude systemic diseases, especially PNS. In addition, the elderly should be screened regularly for tumor markers. Further studies into the PNS mechanisms regulating the immune response are warranted.

## Author contributions

**Conceptualization:** Yuhuan Wang, Sijia Yang.

**Data curation:** Yuhuan Wang, Sijia Yang, Liang Fang, Gang Jiang, Xiaoyan Ding, He Wei.

**Formal analysis:** Yuhuan Wang, Sijia Yang.

**Investigation:** Gang Jiang, Xiaoyan Ding, He Wei.

**Project administration:** Min Liu.

**Resources:** Min Liu.

**Supervision:** Min Liu.

**Validation:** Min Liu.

**Visualization:** Yuhuan Wang, Sijia Yang, Liang Fang, Yang Liu, Min Liu.

**Writing – original draft:** Yuhuan Wang, Sijia Yang.

**Writing – review & editing:** Yuhuan Wang, Sijia Yang, Liang Fang, Yang Liu.
